# Supplementary material for: Historical Assembly of Andean Tree Communities
Source: Plants (Basel). 2023 Oct 12;12(20):3546. doi: 10.3390/plants12203546 (PMC10610186; doi:10.3390/plants12203546)
Supplement: Supplementary file 1 [file plants-12-03546-s001.zip › plants-2448284-SI.pdf]

Supplementary Materials

Methodological remarks

We assessed two potential limitations in our methodology, one related to the simulations and one related to the empirical approach:

1- We used a single combination of the parameter of speciation and extinction in our simulation in the main text that can derive in different phylogenetic tree shapes that also influence phylogenetic diversity estimates. Here, we used multiple combinations of these parameters to assess the influence of different speciation and extinction parameters in our general results.

2- We used a broad taxonomic scale species pool in our empirical analyses, including angiosperms and gymnosperms. Gymnosperms represent long branches in the phylogenetic tree and can affect phylogenetic diversity estimates. Also, gymnosperm species have a low abundance in many of our plots compared to angiosperms.

The phylogenetic diversity patterns can be influenced by the phylogenetic tree's shape, which ultimately depends on the speciation and extinction rates of clades [47,24]. We used different combinations of speciation (0.5, 0.75 and 1) and extinction rates (0.01, 0.05 and 0.1) in our computer simulation to vary the shape of the simulated phylogenetic tree and assess its influence in our conclusions. We did not find any signal of a significant effect of speciation and extinction rates on the patterns of phylogenetic diversity along latitude and elevation neither by the TNC nor by the MZO hypothesis (Fig. S1). Then, our results are consistent with the overall idea that historical dispersal between contrasting biomes had a higher relative importance than speciation or extinction rates on determining the observed patterns of phylogenetic diversity along latitudinal and elevational gradients [48]. Recent studies on plants and vertebrates have found similar results and showed that diversification rates (i.e., speciation minus extinction) are similar across latitude or can even increase poleward (but see [48–50]). These findings support our claims that an increment in the phylogenetic diversity along latitude and elevation could be robust even to variations in the diversification rates.

In South America, the gymnosperms (e.g., Aracauriaceae) were a dominant feature during the Maastrichtian, which may have left its fingerprint on the composition of the subsequent neotropical forest formation after the massive Cretaceous extinction event [20]. A high abundance of gymnosperms could distort or enhance the length of the branches in the phylogenetic tree, and thus, the assessment of the phylogenetic diversity along the latitudinal gradient. Thus, to assess the robustness and validity of our results, we reduced our taxonomic scope to only angiosperms and found that the results remained very much the same than those employing gymnosperms + angiosperms (Fig. S2). These results show that the effect of gymnosperms was not the cause of the pattern of increase of the phylogenetic diversity along with latitude or elevation. (Fig. S2). Then, we confirm that our claims about the MZO hypothesis as an underlying mechanism of the tree community assembly are not an artifact or a particular case on determining the latitudinal and elevational gradient in Andean forests.

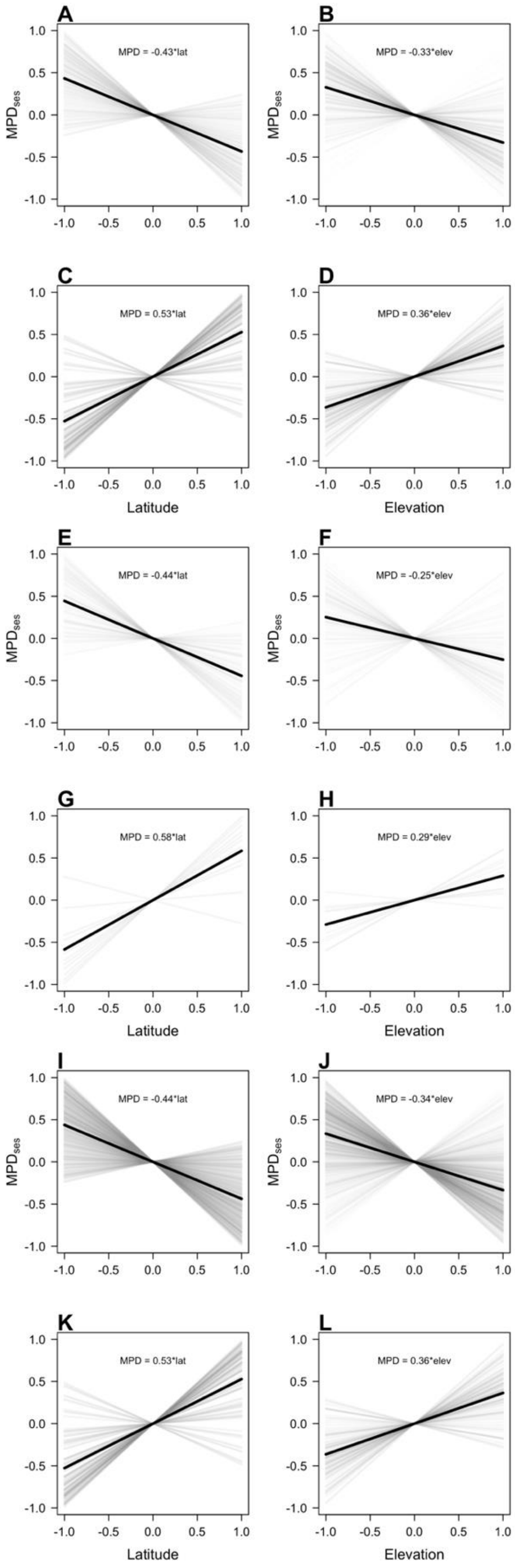

A
MPD = -0.43*lat
B
MPD = -0.33*elev
C
MPD = 0.53*lat
D
MPD = 0.36*elev
Latitude
Elevation
E
MPD = -0.44*lat
F
MPD = -0.25*elev
G
MPD = 0.58*lat
H
MPD = 0.29*elev
Latitude
Elevation
I
MPD = -0.44*lat
J
MPD = -0.34*elev
K
MPD = 0.53*lat
L
MPD = 0.36*elev
Latitude
Elevation
MPD_{ses}

Figure S1. Variation of simulated standardized effect size of mean pairwise phylogenetic distance (MPDses) across latitude and elevation obtained from simulations of the Tropical Niche Conservatism hypothesis (left panels) and the Multiple Zone of Origin hypothesis (right panels). The black lines showed the main effect of the x-axis variable and gray lines represent simulations between 0.25 and 0.75 quantiles. The equation is based on the average of regression coefficients of 1000 simulations. Simulations using speciation rate = 0.5 and extinction = 0.1 (A, B, C, D), simulations using speciation rate = 0.75 and extinction = 0.05 (E, F, G, H) and simulations using speciation rate = 0.5 and extinction = 0.05 (I, J, K, L).

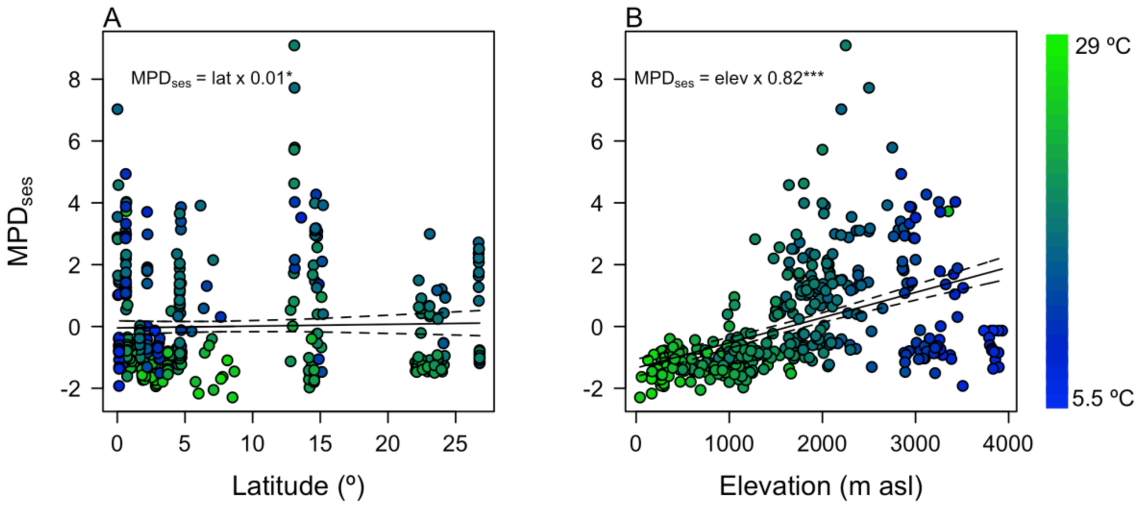

Figure S2. The standardized mean pairwise phylogenetic distance among co-occurring species in each plot (MPD_{ses}) after control by the effect of plot area (i.e., residuals of a regression between MPD_{ses} and plot area) along latitude (A) and elevation (B) including angiosperms alone. The black lines showed the main effect of the variable and the gray lines indicates their confidence intervals. Color indicates mean annual temperature in each plot. Asterisks indicate slope significance.

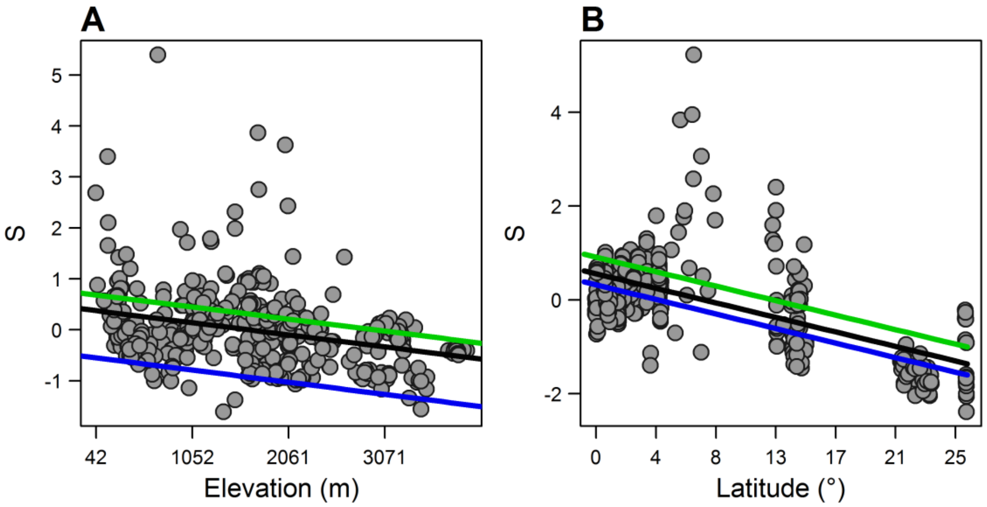

Figure S3. Component plus residuals plots for the species richness in each plot (S) along elevation (A) and latitude (B). The black lines showed the main effect of the variable, and the green and blue lines represent their effect to different values of the other variable.

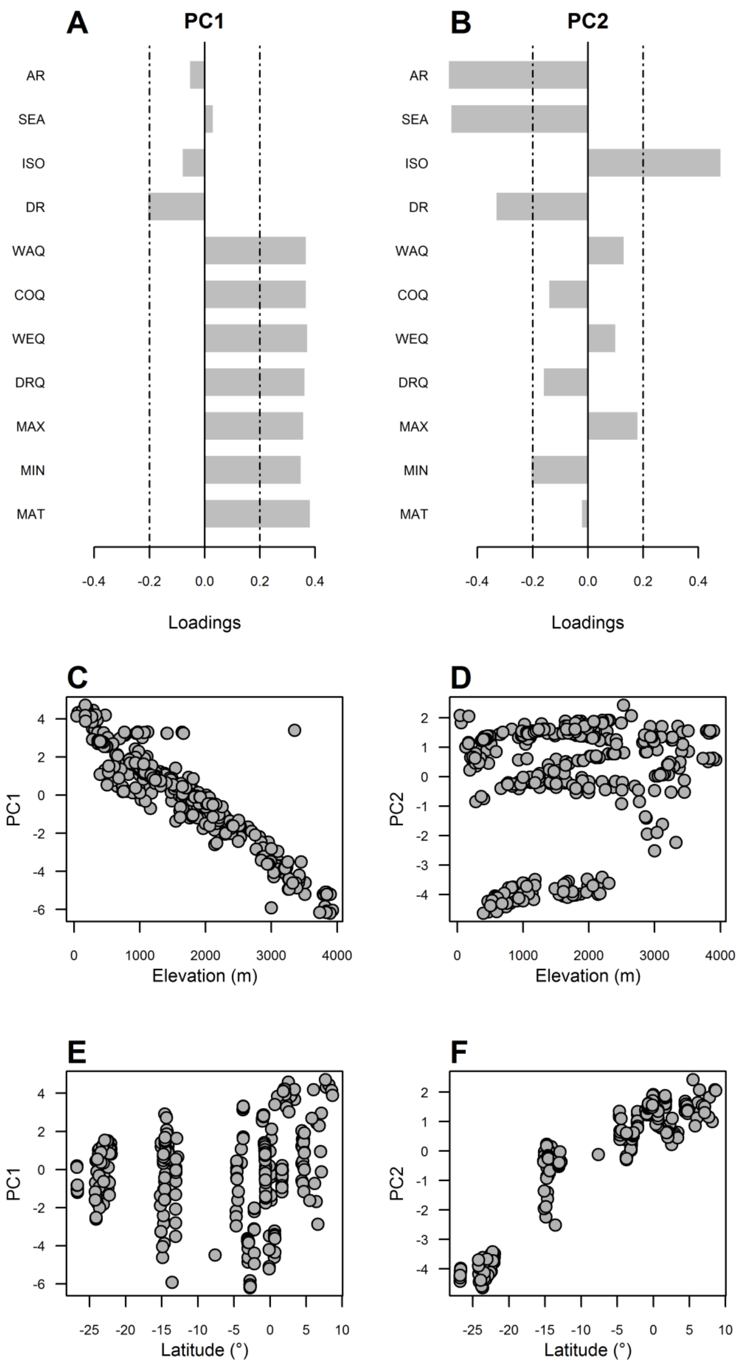

Figure S4. Loadings for the PC1 (A) and PC2 (B) of the principal component analysis of the eleven bioclimatic variables related to temperature used in the Figure 1. The variation of PC1 along elevation (C) and latitudinal (E) gradients. The variation of PC2 along elevation (D) and latitudinal (F) gradients.

Tabla S1. Metadata used in this analyses.

Elev (m)	Long (°)	Lat (°)	Area(m^2)	SR	MPD_OBS	MPD_Rmean	MPD_Rsd	ses.MPD
304	-72,72	1,90	2500,00	53,00	235,56	253,54	15,27	-1,18
330	-72,95	2,09	2500,00	44,00	233,55	251,92	16,10	-1,14
338	-72,92	2,09	2500,00	47,00	233,16	252,52	15,44	-1,25
272	-72,59	2,02	2500,00	56,00	229,86	252,38	14,71	-1,53
276	-72,58	1,03	2500,00	62,00	236,15	252,74	13,58	-1,22
329	-72,58	2,01	2500,00	53,00	235,56	253,26	14,78	-1,20
267	-72,72	1,84	2500,00	66,00	234,51	252,35	13,15	-1,36
232	-72,71	1,82	2500,00	55,00	234,48	252,93	14,85	-1,24
265	-72,71	1,83	2500,00	65,00	233,42	252,86	13,81	-1,41
250	-72,72	1,84	2500,00	61,00	232,08	252,49	13,78	-1,48
312	-72,67	1,97	2500,00	72,00	234,26	252,32	12,61	-1,43
299	-72,72	1,89	2500,00	51,00	228,46	252,85	15,37	-1,59
288	-73,37	2,86	2500,00	65,00	230,06	252,43	13,18	-1,70
248	-73,39	2,88	2500,00	48,00	230,68	252,09	15,63	-1,37
296	-73,40	2,89	2500,00	68,00	240,57	252,84	13,23	-0,93
296	-73,38	2,85	2500,00	49,00	224,77	252,45	15,64	-1,77
278	-73,34	2,85	2500,00	63,00	229,54	251,75	13,12	-1,69
266	-73,36	2,84	2500,00	74,00	230,37	252,80	12,43	-1,81
304	-73,33	2,81	2500,00	50,00	235,03	252,80	16,09	-1,10
261	-73,60	3,39	2500,00	29,00	239,09	252,50	21,18	-0,63
235	-73,38	2,92	2500,00	25,00	222,62	252,44	21,04	-1,42
266	-73,36	2,92	2500,00	35,00	236,36	252,23	17,84	-0,89
285	-72,69	1,89	2500,00	31,00	237,55	253,91	20,09	-0,81
296	-73,39	2,93	2500,00	39,00	222,82	252,04	17,89	-1,63
296	-73,40	2,93	2500,00	34,00	236,05	252,28	18,85	-0,86
304	-73,45	2,84	2500,00	24,00	238,81	252,56	22,99	-0,60
241	-73,42	2,82	2500,00	37,00	224,10	251,42	18,29	-1,49
304	-73,41	2,82	2500,00	47,00	235,87	253,01	15,96	-1,07
279	-74,10	2,19	2500,00	38,00	241,43	252,45	18,69	-0,59
318	-74,08	2,20	2500,00	36,00	227,78	251,49	17,82	-1,33
314	-74,09	2,21	2500,00	51,00	235,72	252,84	15,78	-1,08
359	-74,44	2,28	2500,00	34,00	236,42	252,42	18,57	-0,86
449	-74,43	2,28	2500,00	26,00	234,52	253,55	22,61	-0,84
273	-72,70	1,89	2500,00	34,00	234,23	252,11	19,14	-0,93
510	-74,51	2,61	2500,00	58,00	237,72	252,54	14,16	-1,05
1037	-74,51	2,61	2500,00	33,00	240,73	252,74	19,45	-0,62
312	-72,67	1,97	2500,00	35,00	237,22	252,89	18,75	-0,84
288	-72,78	2,54	2500,00	77,00	234,13	252,42	12,51	-1,46
190	-72,58	2,52	2500,00	49,00	240,89	253,05	15,78	-0,77

473	-72,94	3,35	2500,00	67,00	230,87	252,63	13,53	-1,61
302	-72,84	1,98	2500,00	71,00	239,55	252,62	13,11	-1,00
234	-72,83	1,97	2500,00	65,00	233,69	252,34	13,50	-1,38
203	-72,82	1,97	2500,00	42,00	231,82	252,95	17,17	-1,23
234	-72,83	1,96	2500,00	46,00	231,36	252,56	15,95	-1,33
349	-72,92	2,07	2500,00	36,00	226,08	252,29	17,85	-1,47
772	-80,63	-3,77	2500,00	38,00	232,47	252,71	18,48	-1,10
654	-64,57	-24,13	10000,00	32,00	233,03	252,15	19,11	-1,00
1181	-63,90	-22,02	10000,00	41,00	236,81	252,84	16,81	-0,95
1014	-63,92	-22,04	10000,00	44,00	232,71	251,88	15,78	-1,21
465	-64,47	-23,12	10000,00	51,00	234,79	252,49	15,53	-1,14
439	-77,49	-1,07	3600,00	31,00	242,91	251,94	19,01	-0,48
473	-77,50	-1,07	3600,00	42,00	243,02	252,47	16,12	-0,59
407	-77,50	-1,06	3600,00	37,00	241,56	251,64	17,46	-0,58
735	-64,94	-24,01	10000,00	39,00	231,08	252,32	17,54	-1,21
2027	-75,70	6,15	10000,00	233,00	281,81	252,79	6,91	4,20
1740	-75,14	6,99	10000,00	240,00	251,51	253,03	7,05	-0,22
1984	-64,85	-23,06	10000,00	19,00	275,66	253,12	25,89	0,87
749	-80,64	-3,78	2500,00	65,00	234,90	252,44	13,99	-1,25
1684	-64,74	-22,46	10000,00	33,00	265,31	252,99	19,56	0,63
1973	-64,74	-22,46	10000,00	22,00	279,71	253,26	23,74	1,11
2928	-75,65	6,61	10000,00	80,00	271,58	252,60	12,28	1,55
2134	-64,85	-23,09	10000,00	4,00	437,46	253,29	57,17	3,22
829	-63,90	-22,13	10000,00	43,00	232,69	251,80	16,49	-1,16
778	-63,90	-22,10	10000,00	38,00	233,93	251,94	17,69	-1,02
1696	-65,36	-26,72	2400,00	6,00	375,62	253,06	45,74	2,68
1752	-65,34	-26,70	4800,00	9,00	320,55	252,07	36,30	1,89

1607	-65,34	-26,71	3200,00	14,00	282,29	253,37	30,42	0,95
1644	-65,35	-26,72	2400,00	9,00	323,77	252,86	36,55	1,94
1643	-65,35	-26,71	2400,00	7,00	347,50	251,88	41,43	2,31
1871	-65,34	-26,70	2400,00	5,00	380,66	252,04	49,93	2,58
1755	-65,34	-26,70	1600,00	6,00	384,85	252,25	44,27	3,00
1792	-65,34	-26,70	2400,00	6,00	362,04	251,81	44,12	2,50
1672	-65,36	-26,73	2400,00	6,00	375,62	252,70	45,79	2,68
1757	-65,33	-26,70	2400,00	11,00	300,75	251,42	32,55	1,52
941	-80,67	-3,75	2500,00	53,00	242,97	253,08	15,58	-0,65
2646	-76,03	6,38	10000,00	147,00	260,03	252,91	8,73	0,82
1500	-71,78	-12,81	10000,00	151,00	259,46	252,37	8,56	0,83
1250	-71,78	-12,80	10000,00	141,00	244,50	252,17	9,23	-0,83
59	-76,76	7,78	10000,00	122,00	238,92	252,60	10,04	-1,36
128	-74,94	8,13	10000,00	109,00	243,70	252,81	10,17	-0,90
1136	-64,75	-23,09	10000,00	40,00	237,86	252,29	17,22	-0,84
572	-64,45	-22,63	10000,00	36,00	234,10	251,89	17,54	-1,01
805	-65,33	-26,77	12800,00	27,00	232,97	253,87	22,12	-0,94
3450	-77,48	-7,64	10000,00	28,00	264,00	251,39	19,33	0,65
619	-64,14	-22,92	10000,00	42,00	231,94	252,78	17,27	-1,21
1500	-79,97	-3,99	10000,00	45,00	264,70	252,73	16,68	0,72
2140	-65,49	-24,11	10000,00	4,00	235,75	254,94	63,71	-0,30
1119	-80,67	-3,72	2500,00	71,00	236,82	252,67	13,26	-1,19
972	-80,66	-3,74	2500,00	54,00	241,35	252,29	14,66	-0,75

3511	-77,70	0,59	3600,00	17,00	290,89	254,23	28,24	1,30
3410	-77,70	0,59	3600,00	13,00	310,27	252,76	31,04	1,85
3037	-77,84	0,36	3600,00	21,00	231,33	252,10	23,42	-0,89
3352	-79,49	0,66	3600,00	16,00	360,82	254,76	29,62	3,58
3428	-77,64	0,66	3600,00	14,00	377,44	251,84	28,02	4,48
2313	-78,69	-0,02	3600,00	18,00	349,78	253,07	26,82	3,61
2282	-78,69	-0,01	3600,00	28,00	316,39	252,67	21,24	3,00
2203	-78,69	-0,01	3600,00	16,00	452,43	253,77	27,74	7,16
2492	-78,57	0,12	3600,00	26,00	287,34	252,61	21,88	1,59
2212	-78,57	0,11	3600,00	18,00	296,67	250,83	24,37	1,88
1879	-78,72	0,05	3600,00	18,00	300,19	253,41	26,00	1,80
1829	-78,72	0,05	3600,00	28,00	277,40	252,20	20,01	1,26
827	-78,88	0,16	3600,00	15,00	243,35	252,09	27,47	-0,32
1018	-78,53	0,10	3600,00	10,00	244,76	249,76	30,29	-0,17
653	-78,91	0,19	3600,00	15,00	236,79	251,46	27,86	-0,53
632	-78,91	0,19	3600,00	20,00	246,07	252,15	24,68	-0,25
1277	-78,81	-0,03	3600,00	33,00	310,17	252,36	19,00	3,04
1640,3	-78,74	-0,08	3600,00	48,00	328,01	253,42	15,73	4,74
3421,4	-78,60	-0,12	3600,00	25,00	279,46	253,13	22,11	1,19
2932	-78,60	-0,10	3600,00	18,00	236,30	254,27	27,25	-0,66
3109	-78,60	-0,10	3600,00	21,00	241,87	255,19	25,96	-0,51
3507	-78,59	-0,13	3600,00	9,00	187,33	251,04	36,16	-1,76
166	-74,82	7,66	10000,00	220,00	242,10	252,61	7,20	-1,46
1548	-65,10	-24,12	10000,00	28,00	266,65	253,29	21,81	0,61
1065	-80,68	-3,72	2500,00	50,00	237,58	252,50	15,57	-0,96
1084	-65,06	-23,99	10000,00	37,00	237,19	252,14	17,48	-0,85
1412	-80,69	-3,72	2500,00	76,00	239,86	252,24	12,37	-1,00
741	-65,32	-26,77	9600,00	28,00	239,81	252,67	21,20	-0,61
1414	-80,70	-3,71	2500,00	48,00	235,23	251,91	15,39	-1,08
1159	-65,14	-24,19	10000,00	30,00	238,26	252,33	19,77	-0,71
1642	-80,70	-3,72	2500,00	71,00	236,99	253,34	14,11	-1,16
1106	-73,51	4,38	2500,00	44,00	233,92	252,58	16,57	-1,13
1304	-73,51	4,39	2500,00	36,00	238,44	253,25	18,96	-0,78
1800	-73,53	4,40	2500,00	19,00	238,84	253,39	25,53	-0,57
2059	-73,54	4,40	2500,00	20,00	224,64	252,61	24,81	-1,13
1066	-73,44	4,48	2500,00	36,00	237,61	252,67	18,99	-0,79
1370	-73,45	4,50	2500,00	36,00	230,16	251,97	17,28	-1,26
1751	-73,45	4,50	2500,00	29,00	240,77	252,01	19,72	-0,57

2072	-73,46	4,50	2500,00	43,00	234,67	252,14	16,51	-1,06
1068	-73,39	4,61	2500,00	40,00	236,92	252,56	17,84	-0,88
1543	-73,41	4,62	2500,00	37,00	238,64	252,89	17,85	-0,80
1889	-73,42	4,62	2500,00	23,00	230,76	254,15	24,79	-0,94
2275	-73,45	4,66	2500,00	30,00	264,55	252,76	20,47	0,58
3372	-79,17	-3,06	500,00	20,00	233,24	252,03	24,52	-0,77
3385	-79,16	-3,06	500,00	21,00	230,61	251,49	22,97	-0,91
3290	-79,16	-3,07	500,00	27,00	235,97	252,85	20,79	-0,81
3218	-79,15	-3,07	500,00	22,00	235,70	252,59	23,38	-0,72
3011	-79,11	-3,08	500,00	24,00	243,90	252,43	21,99	-0,39
3100	-79,12	-3,08	500,00	17,00	235,28	251,97	26,70	-0,63
3050	-79,11	-3,08	500,00	25,00	238,25	254,01	22,99	-0,69
1068	-73,34	4,73	2500,00	41,00	234,37	252,40	17,38	-1,04
1483	-73,36	4,74	2500,00	18,00	235,73	251,88	25,06	-0,64
1810	-73,37	4,74	2500,00	37,00	237,47	254,23	19,05	-0,88
2013	-73,39	4,75	2500,00	28,00	238,16	253,28	21,76	-0,69
1663	-80,70	-3,71	2500,00	57,00	232,85	252,43	14,72	-1,33
2527	-75,90	5,49	10000,00	121,00	253,44	252,41	9,65	0,11
1848	-80,72	-3,69	2500,00	50,00	237,99	252,78	15,53	-0,95
845	-63,84	-22,27	10000,00	47,00	235,71	252,53	16,45	-1,02
827	-63,88	-22,22	10000,00	44,00	235,58	252,70	16,14	-1,06
990	-63,95	-22,13	10000,00	46,00	232,62	253,47	16,60	-1,26
1903	-80,72	-3,68	2500,00	47,00	282,13	252,65	15,73	1,87
910	-65,33	-26,76	11200,00	25,00	238,91	251,77	22,06	-0,58
886	-65,33	-26,76	10800,00	24,00	235,30	252,26	21,84	-0,78
1486	-64,80	-23,08	10000,00	34,00	263,31	252,64	19,08	0,56
1175	-64,73	-22,44	10000,00	55,00	253,04	252,26	14,67	0,05
1380	-80,14	-3,98	10000,00	36,00	239,95	253,81	18,77	-0,74
473	-64,46	-23,77	10000,00	34,00	232,95	252,52	18,73	-1,04
2111	-80,72	-3,68	2500,00	45,00	237,38	253,54	16,40	-0,99
982	-64,74	-23,09	10000,00	42,00	263,37	252,22	16,82	0,66

928	-74,79	6,46	10000,00	168,00	247,54	252,97	8,42	-0,65
1082	-64,87	-23,70	10000,00	38,00	238,35	252,45	17,52	-0,80
1747	-64,90	-23,68	10000,00	27,00	262,82	252,93	21,56	0,46
740	-65,32	-26,77	10000,00	25,00	240,95	253,42	22,91	-0,54
996	-63,94	-22,21	10000,00	39,00	237,80	251,84	17,88	-0,79
2884	-78,44	-2,23	400,00	9,00	368,97	254,59	39,46	2,90
2530	-78,36	-2,19	400,00	12,00	215,01	251,46	31,19	-1,17
3266	-78,48	-2,20	400,00	6,00	429,74	255,05	49,47	3,53
3261	-78,48	-2,20	400,00	13,00	312,08	252,81	30,85	1,92
3268	-78,48	-2,20	400,00	6,00	225,42	249,94	41,90	-0,59
3257	-78,48	-2,20	400,00	10,00	242,43	252,77	34,55	-0,30
3270	-78,48	-2,20	400,00	5,00	260,13	250,24	47,55	0,21
2873	-78,44	-2,23	400,00	15,00	308,62	251,12	27,44	2,10
2866	-78,44	-2,22	400,00	15,00	311,54	254,02	30,18	1,91
2859	-78,46	-2,20	400,00	18,00	305,31	253,19	26,79	1,95
2878	-78,45	-2,20	400,00	16,00	232,85	254,72	30,56	-0,72
2296	-78,36	-2,20	400,00	22,00	241,91	252,69	22,94	-0,47
2399	-78,37	-2,20	400,00	21,00	289,62	254,18	26,26	1,35
2361	-78,37	-2,20	400,00	23,00	239,21	252,47	22,72	-0,58
2385	-78,37	-2,20	400,00	23,00	297,67	254,04	22,99	1,90
983	-77,59	-0,82	3600,00	36,00	242,53	252,82	18,41	-0,56
975	-77,59	-0,81	3600,00	52,00	238,05	252,96	15,49	-0,96
1000	-77,58	-0,82	3600,00	49,00	241,21	252,32	15,25	-0,73
2083	-80,73	-3,68	2500,00	40,00	261,86	253,11	17,34	0,50
2092	-77,84	-0,63	400,00	22,00	288,48	252,63	23,28	1,54
1802,476	-77,80	-0,65	400,00	37,00	327,49	253,09	17,89	4,16
1475,79	-77,78	-0,64	400,00	14,00	322,16	251,37	28,54	2,48
1602,401	-77,78	-0,64	400,00	31,00	241,36	252,30	19,21	-0,57
1721,352	-77,79	-0,65	400,00	31,00	314,42	253,12	20,34	3,01
1769,153	-77,79	-0,65	400,00	37,00	273,72	251,90	17,44	1,25
1777,564	-77,79	-0,65	400,00	34,00	276,93	252,88	18,58	1,29
2363,924	-77,82	-0,62	400,00	19,00	334,96	254,96	26,63	3,00

2382,861	-77,82	-0,62	400,00	19,00	298,34	253,28	26,17	1,72
2300,343	-77,82	-0,62	400,00	20,00	237,43	253,32	26,06	-0,61
2272,7	-77,82	-0,62	400,00	28,00	283,24	254,32	22,07	1,31
2161,966	-77,84	-0,63	400,00	20,00	292,95	252,25	24,47	1,66
2335,539	-77,83	-0,62	400,00	25,00	282,58	254,67	23,42	1,19
2415,963	-77,83	-0,62	400,00	18,00	290,97	252,86	25,55	1,49
2067,867	-77,84	-0,63	400,00	14,00	316,06	255,50	31,92	1,90
2078,959	-77,84	-0,64	400,00	19,00	300,61	252,75	25,15	1,90
2006,23	-77,84	-0,64	400,00	28,00	284,30	253,25	20,68	1,50
1880,666	-77,80	-0,65	400,00	23,00	284,96	252,43	22,70	1,43
1917,916	-77,80	-0,65	400,00	32,00	300,70	253,10	19,03	2,50
1910,965	-77,80	-0,65	400,00	24,00	281,36	252,76	21,72	1,32
1873,148	-77,80	-0,65	400,00	29,00	266,74	252,56	20,33	0,70
41	-76,66	8,51	10000,00	190,00	236,55	252,55	7,76	-2,06
1650	-64,72	-22,28	10000,00	19,00	274,63	252,42	25,99	0,85
2200	-64,75	-22,27	10000,00	12,00	308,24	252,33	33,40	1,67
751	-65,33	-26,77	6000,00	26,00	231,52	252,17	20,65	-1,00
2364	-80,73	-3,68	2500,00	29,00	273,34	252,82	20,70	0,99
1961	-65,28	-23,97	10000,00	15,00	294,09	252,10	28,78	1,46
2314	-80,73	-3,68	2500,00	37,00	255,07	253,30	18,77	0,09
1937	-76,07	1,67	2500,00	11,00	239,79	254,27	35,43	-0,41
1949	-76,07	1,68	2500,00	3,00	224,37	254,15	69,95	-0,43
1859	-76,07	1,69	2500,00	9,00	221,15	251,94	36,04	-0,85
2018	-76,06	1,68	2500,00	4,00	263,47	252,90	57,68	0,18
1810	-76,06	1,66	2500,00	8,00	234,51	253,99	40,72	-0,48
2035	-76,06	1,68	2500,00	17,00	243,18	253,30	28,04	-0,36
1793	-76,08	1,67	2500,00	10,00	234,58	252,10	34,20	-0,51

1980	-76,06	1,67	2500,00	4,00	255,45	253,61	59,77	0,03
1721	-76,07	1,66	2500,00	11,00	245,65	252,72	33,83	-0,21
1682	-76,08	1,66	2500,00	3,00	213,45	250,74	62,02	-0,60
1639	-76,09	1,65	2500,00	5,00	242,24	249,56	45,00	-0,16
1676	-76,10	1,64	2500,00	15,00	236,57	252,27	29,82	-0,53
1896	-76,09	1,64	2500,00	19,00	241,31	254,16	26,02	-0,49
1941	-76,09	1,64	2500,00	9,00	243,51	252,45	36,35	-0,25
1793	-76,09	1,63	2500,00	26,00	239,85	253,33	22,07	-0,61
1962	-76,09	1,63	2500,00	9,00	243,02	251,86	35,47	-0,25
1701	-76,02	1,64	2500,00	9,00	238,86	249,59	32,26	-0,33
1929	-76,07	1,67	2500,00	8,00	246,85	250,76	37,57	-0,10
1658	-76,03	1,65	2500,00	7,00	235,16	252,76	43,38	-0,41
1788	-76,03	1,64	2500,00	7,00	249,95	251,77	40,55	-0,04
2204	-76,28	1,63	2500,00	22,00	246,75	252,70	23,10	-0,26
2077	-76,28	1,63	2500,00	30,00	243,94	252,85	20,71	-0,43
2072	-76,27	1,64	2500,00	17,00	246,67	252,37	27,40	-0,21
2909	-79,46	-4,71	3600,00	11,00	305,53	251,70	32,07	1,68
2955	-79,46	-4,71	3600,00	3,00	524,01	257,22	74,17	3,60
2817	-79,46	-4,70	3600,00	5,00	420,44	254,90	51,76	3,20
2840	-79,46	-4,70	3600,00	4,00	456,90	254,23	58,10	3,49
3049	-79,46	-4,71	3600,00	3,00	256,11	252,76	67,52	0,05
3096	-79,46	-4,71	3600,00	4,00	225,89	252,86	58,26	-0,46
2304	-64,93	-23,63	10000,00	15,00	292,95	252,74	28,29	1,42
3210	-78,98	-3,14	500,00	18,00	216,86	252,27	25,90	-1,37
3100	-78,98	-3,14	500,00	21,00	231,04	254,57	24,97	-0,94
3151	-78,99	-3,13	500,00	19,00	246,40	252,58	26,50	-0,23
3120	-78,99	-3,12	500,00	28,00	238,69	252,49	20,78	-0,66
3083	-78,99	-3,12	500,00	24,00	232,47	252,55	22,52	-0,89
977	-75,08	6,78	10000,00	120,00	248,88	252,65	10,14	-0,37
835	-68,66	-14,20	10000,00	55,00	234,24	252,60	14,82	-1,24
935	-68,65	-14,20	10000,00	56,00	234,15	252,72	14,50	-1,28
1150	-68,64	-14,20	10000,00	96,00	233,27	252,33	11,37	-1,68
400	-67,92	-14,42	10000,00	116,00	242,40	252,90	10,18	-1,03
2872	-69,02	-14,68	10000,00	38,00	313,74	252,70	17,65	3,46
3116	-69,03	-14,69	10000,00	34,00	337,88	253,82	19,43	4,33

1232	-68,73	-14,76	10000,00	68,00	252,65	252,63	13,27	0,00
850	-68,59	-14,25	10000,00	55,00	233,68	252,69	14,93	-1,27
1890	-68,25	-14,50	10000,00	78,00	280,17	252,67	12,82	2,15
1247	-68,71	-14,67	10000,00	100,00	238,85	252,52	11,12	-1,23
2000	-68,93	-14,60	10000,00	70,00	308,02	253,48	13,27	4,11
1900	-68,93	-14,60	10000,00	85,00	272,89	252,46	11,66	1,75
280	-67,65	-14,61	10000,00	124,00	239,67	252,48	9,84	-1,30
795	-68,52	-14,25	10000,00	52,00	230,18	251,64	14,49	-1,48
933	-68,53	-14,25	10000,00	65,00	234,34	252,76	13,89	-1,33
2766	-68,99	-14,77	10000,00	36,00	310,54	253,24	18,58	3,08
3324	-68,86	-14,89	10000,00	16,00	297,02	254,24	28,64	1,49
1522	-68,65	-14,79	10000,00	95,00	259,74	252,13	10,68	0,71
1538	-68,64	-14,77	10000,00	98,00	283,50	252,66	10,59	2,91
1242	-68,47	-15,08	10000,00	137,00	248,59	253,18	9,28	-0,49
1054	-68,46	-15,09	10000,00	105,00	265,31	252,29	10,03	1,30
1626	-68,20	-14,43	10000,00	83,00	275,62	253,26	12,07	1,85
880	-68,54	-14,47	10000,00	50,00	232,40	251,82	15,49	-1,25
662	-68,57	-14,33	10000,00	50,00	234,60	252,61	15,32	-1,18
840	-68,57	-14,33	10000,00	42,00	234,70	253,13	17,25	-1,07
1020	-68,57	-14,33	10000,00	61,00	233,19	252,99	14,86	-1,33
1100	-68,72	-14,16	10000,00	88,00	235,44	252,43	11,94	-1,42

1250	-68,75	-14,15	10000,00	90,00	239,01	252,86	11,68	-1,19
1450	-68,74	-14,16	10000,00	88,00	243,61	252,79	11,64	-0,79
2498	-68,97	-14,78	10000,00	40,00	310,33	253,39	17,54	3,25
1005	-68,78	-14,58	10000,00	71,00	234,97	252,66	13,18	-1,34
1223	-68,78	-14,57	10000,00	94,00	238,65	252,64	10,86	-1,29
1993	-68,94	-14,74	10000,00	92,00	269,63	252,79	11,14	1,51
1804	-68,95	-14,75	10000,00	82,00	252,55	253,37	11,82	-0,07
2697	-68,76	-15,21	10000,00	43,00	322,49	253,88	17,41	3,94
2369	-68,76	-15,20	10000,00	44,00	263,19	253,80	17,45	0,54
2889	-68,92	-15,11	10000,00	34,00	229,31	252,50	18,44	-1,26
1400	-68,59	-14,78	10000,00	111,00	249,52	252,86	10,46	-0,32
1468	-68,58	-14,79	10000,00	90,00	252,73	252,61	11,67	0,01
2859	-68,88	-15,13	10000,00	35,00	296,26	253,15	18,96	2,27
2200	-68,95	-14,61	10000,00	79,00	291,99	252,92	12,60	3,10
2400	-68,95	-14,62	10000,00	81,00	293,28	253,61	11,92	3,33
2510	-68,96	-14,63	10000,00	60,00	300,96	252,56	13,91	3,48
362	-67,96	-14,36	10000,00	127,00	251,08	252,67	10,17	-0,16
3035	-68,85	-14,89	10000,00	23,00	233,69	251,65	22,87	-0,79
1000	-68,69	-14,54	10000,00	48,00	227,82	253,26	16,72	-1,52
1180	-68,69	-14,55	10000,00	56,00	230,59	253,17	15,20	-1,49
850	-68,69	-14,55	10000,00	40,00	232,22	251,81	17,00	-1,15

167	-74,61	6,01	10000,0<br>0	157,0<br>0	235,95	252,53	8,66	-1,92
2647	-80,74	-3,68	2500,00	34,00	229,34	252,22	18,71	-1,22
2802	-80,75	-3,67	2500,00	20,00	228,43	253,04	25,03	-0,98
2166	-65,48	-<br>24,10	10000,0<br>0	5,00	236,89	251,31	48,43	-0,30
596	-63,97	-<br>22,45	10000,0<br>0	34,00	237,15	253,52	19,91	-0,82
711	-63,93	-<br>22,55	10000,0<br>0	45,00	235,91	252,72	16,78	-1,00
960	-80,65	-3,75	2500,00	53,00	232,63	251,74	14,73	-1,30
609	-64,04	-<br>22,57	10000,0<br>0	36,00	231,39	252,52	18,38	-1,15
453	-77,31	-0,98	3600,00	47,00	236,38	252,46	15,20	-1,06
395	-77,31	-0,98	3600,00	34,00	237,10	252,69	19,28	-0,81
392	-77,31	-0,98	3600,00	53,00	234,99	253,19	15,56	-1,17
360	-77,24	-0,90	3600,00	32,00	239,10	251,93	19,60	-0,65
376	-77,24	-0,90	3600,00	39,00	242,62	253,09	18,31	-0,57
381	-77,24	-0,90	3600,00	39,00	241,77	252,22	17,15	-0,61
3232	-78,86	-2,99	500,00	13,00	237,41	252,54	30,18	-0,50
3196	-78,87	-2,99	500,00	13,00	230,17	252,54	31,48	-0,71
3210	-78,87	-3,00	500,00	12,00	234,97	252,75	32,25	-0,55
171	-77,36	8,65	10000,0<br>0	167,0<br>0	242,46	252,58	8,54	-1,19
396	-64,54	-<br>23,65	10000,0<br>0	24,00	237,10	251,94	22,19	-0,67
691	-74,73	7,11	10000,0<br>0	299,0<br>0	241,49	252,62	6,26	-1,78
1051	-64,56	-<br>22,25	10000,0<br>0	44,00	240,20	253,74	17,21	-0,79
595	-64,80	-<br>23,77	10000,0<br>0	28,00	228,90	251,68	19,97	-1,14
1750	-71,54	-<br>13,05	10000,0<br>0	180,0<br>0	268,44	252,96	7,99	1,94
1500	-71,54	-<br>13,05	10000,0<br>0	163,0<br>0	261,48	252,36	8,37	1,09
2884	-77,50	0,62	400,00	22,00	325,15	252,21	23,13	3,15
2939	-77,52	0,63	400,00	18,00	357,56	253,21	26,15	3,99
2884	-77,50	0,62	400,00	15,00	287,64	252,29	28,67	1,23
2898	-77,51	0,61	400,00	24,00	235,09	252,53	22,69	-0,77
2887	-77,50	0,61	400,00	13,00	221,30	251,99	31,11	-0,99
2846	-77,50	0,62	400,00	19,00	381,61	252,40	24,25	5,33
2979	-77,55	0,62	400,00	22,00	323,55	253,05	23,81	2,96

2992	-77,55	0,62	400,00	27,00	316,19	253,25	21,10	2,98
2962	-77,52	0,63	400,00	20,00	291,37	253,30	25,32	1,50
2935	-77,52	0,63	400,00	20,00	338,47	253,44	25,11	3,39
1000	-65,33	-26,76	10000,00	32,00	237,81	252,22	18,65	-0,77
1000	-65,33	-26,76	10000,00	26,00	236,45	252,59	21,69	-0,74
1000	-65,33	-26,76	10000,00	26,00	240,29	252,85	21,38	-0,59
1000	-65,33	-26,76	10000,00	30,00	235,43	252,68	20,16	-0,86
1000	-65,33	-26,76	10000,00	25,00	237,06	252,36	22,03	-0,69
1000	-65,33	-26,76	10000,00	29,00	236,52	252,36	20,72	-0,76
764	-80,65	-3,74	2500,00	47,00	241,64	252,43	16,15	-0,67
521	-64,46	-23,22	10000,00	44,00	232,82	252,28	16,98	-1,15
586	-75,67	5,78	10000,00	70,00	232,29	251,70	12,71	-1,53
524	-64,45	-22,93	10000,00	42,00	234,41	252,66	16,58	-1,10
1616	-64,79	-23,07	10000,00	31,00	270,26	252,30	18,56	0,97
1000	-71,57	-12,96	10000,00	140,00	254,90	252,84	9,19	0,22
3811	-79,21	-2,78	100,00	1,00	NA	NA	NA	NA
3841	-79,21	-2,77	100,00	1,00	NA	NA	NA	NA
3930	-79,22	-2,78	100,00	1,00	NA	NA	NA	NA
3890	-79,23	-2,78	100,00	1,00	NA	NA	NA	NA
3833	-79,22	-2,77	100,00	1,00	NA	NA	NA	NA
3735	-79,22	-2,77	100,00	1,00	NA	NA	NA	NA
3450	-71,61	-13,11	10000,00	34,00	292,75	253,44	20,07	1,96
3250	-71,61	-13,11	10000,00	46,00	321,81	252,96	16,16	4,26
3000	-71,60	-13,11	10000,00	37,00	296,07	253,67	18,38	2,31
2750	-71,59	-13,11	10000,00	54,00	342,74	253,04	15,02	5,97
2500	-71,57	-13,09	10000,00	58,00	367,39	252,74	14,08	8,14

2250	-71,57	-13,08	10000,00	82,00	365,38	253,13	12,04	9,33
2000	-71,56	-13,07	10000,00	80,00	325,55	253,47	12,55	5,74
1800	-71,56	-13,07	10000,00	114,00	302,27	252,82	10,14	4,88
498	-77,68	-1,06	3600,00	45,00	242,42	252,44	16,74	-0,60
488	-77,68	-1,06	3600,00	25,00	231,44	253,44	22,97	-0,96
458	-77,68	-1,05	3600,00	41,00	240,97	252,13	16,06	-0,69
2056	-75,48	7,08	10000,00	184,00	271,64	252,69	7,90	2,40
677	-64,52	-23,44	10000,00	52,00	233,43	252,35	15,43	-1,23
934	-64,67	-23,44	10000,00	38,00	234,37	252,17	17,32	-1,03
441	-77,27	-0,92	3600,00	34,00	245,12	252,19	18,57	-0,38
414	-77,28	-0,92	3600,00	31,00	235,18	251,79	18,94	-0,88
396	-77,28	-0,92	3600,00	37,00	243,68	251,75	17,79	-0,45
1144	-77,63	-0,73	800,00	28,00	242,64	253,20	21,49	-0,49
1249	-77,61	-0,70	800,00	43,00	239,32	251,42	16,26	-0,74
1123	-77,66	-0,75	800,00	37,00	243,30	253,03	18,40	-0,53
1057	-77,65	-0,75	800,00	45,00	266,39	253,09	16,16	0,82
1054	-77,65	-0,75	800,00	35,00	240,75	253,02	18,95	-0,65
1071	-77,65	-0,75	800,00	34,00	247,45	252,43	17,85	-0,28
1089	-77,64	-0,75	800,00	27,00	248,25	252,90	21,78	-0,21
1135	-77,63	-0,73	800,00	18,00	214,25	253,54	26,72	-1,47
1158	-77,63	-0,73	800,00	47,00	238,76	252,78	16,19	-0,87
1149	-77,64	-0,73	800,00	47,00	242,45	252,55	15,95	-0,63
1400	-77,63	-0,70	800,00	45,00	240,55	252,44	16,15	-0,74
1314	-77,63	-0,69	800,00	29,00	237,88	252,71	21,06	-0,70
1301	-77,62	-0,69	800,00	38,00	241,12	252,19	17,67	-0,63
1127	-77,64	-0,74	800,00	45,00	245,29	252,12	16,27	-0,42
1316	-77,62	-0,69	800,00	41,00	240,30	253,16	16,94	-0,76
1130	-77,61	-0,73	800,00	36,00	237,66	253,32	18,48	-0,85
1074	-77,59	-0,74	800,00	53,00	244,76	252,88	15,04	-0,54
1166	-77,61	-0,73	800,00	49,00	243,00	253,26	15,91	-0,64
1200	-77,63	-0,71	800,00	25,00	234,28	253,02	22,63	-0,83
1232	-77,63	-0,70	800,00	30,00	239,65	253,53	20,95	-0,66
3000	-71,59	-13,59	10000,00	55,00	308,33	253,28	14,81	3,72
3790	-78,59	-0,13	100,00	6,00	242,36	252,35	46,63	-0,21
3857	-78,59	-0,13	100,00	8,00	215,38	251,65	39,22	-0,92
3891	-78,58	-0,13	100,00	8,00	224,61	253,43	42,69	-0,68

3894	-78,58	-0,13	100,00	9,00	208,68	253,38	37,73	-1,18
3798	-78,59	-0,13	100,00	9,00	235,20	253,84	38,26	-0,49
3835,8	-78,59	-0,13	100,00	8,00	227,15	252,99	40,93	-0,63
3820,6	-78,59	-0,13	100,00	10,00	232,20	253,24	36,38	-0,58
3820,4	-78,59	-0,13	100,00	8,00	227,33	252,94	40,05	-0,64
3833,4	-78,58	-0,13	100,00	6,00	195,85	254,24	48,00	-1,22
3843	-78,58	-0,13	100,00	5,00	218,01	252,14	49,19	-0,69
500	-64,91	-23,94	10000,00	31,00	229,70	252,89	20,02	-1,16
1610	-65,08	-24,24	10000,00	19,00	283,16	252,64	25,70	1,19
2059	-65,06	-24,23	10000,00	16,00	285,00	252,04	27,68	1,19
1853	-78,92	-4,61	400,00	26,00	279,33	253,55	22,29	1,16
2107	-78,91	-4,61	400,00	39,00	266,39	252,61	17,66	0,78
1692	-78,94	-4,62	400,00	34,00	235,58	252,81	18,87	-0,91
1659	-78,96	-4,62	400,00	34,00	268,97	253,17	18,25	0,87
1649	-78,96	-4,62	400,00	32,00	238,09	252,43	18,74	-0,77
1697	-78,95	-4,62	400,00	23,00	234,02	251,61	20,84	-0,84
1644	-78,96	-4,62	400,00	33,00	238,63	251,78	17,96	-0,73
2060	-78,90	-4,61	400,00	16,00	308,48	253,92	28,07	1,94
2067	-78,90	-4,61	400,00	15,00	314,68	252,84	28,70	2,15
2016	-78,90	-4,61	400,00	14,00	365,34	252,84	29,00	3,88
2047	-78,90	-4,61	400,00	14,00	317,48	253,39	30,53	2,10
1835	-78,92	-4,62	400,00	21,00	288,06	252,71	23,42	1,51
2083	-78,91	-4,61	400,00	19,00	297,34	252,39	24,70	1,82
1829	-78,92	-4,62	400,00	26,00	281,26	253,71	21,81	1,26
1912	-78,92	-4,61	400,00	25,00	286,14	253,70	22,49	1,44
1918	-78,92	-4,61	400,00	31,00	269,98	253,12	20,23	0,83
2094	-78,91	-4,61	400,00	26,00	244,25	252,69	22,08	-0,38
2035	-78,91	-4,61	400,00	26,00	280,83	251,43	20,04	1,47
2157	-78,91	-4,60	400,00	34,00	271,40	252,13	18,78	1,03
2108	-78,91	-4,61	400,00	21,00	289,42	253,78	25,06	1,42
2419	-79,12	-4,49	10000,00	83,00	259,50	253,69	12,77	0,46
2109	-79,06	-4,41	10000,00	153,00	275,29	252,52	8,70	2,62
928	-78,62	-3,73	10000,00	92,00	239,20	253,11	11,19	-1,24
1055	-78,62	-3,73	10000,00	100,00	238,33	252,79	10,66	-1,36

References

47. M. Vellend, W. K. Cornwell, K. Magnuson-Ford, A. Ø. Mooers, Measuring phylogenetic biodiversity. *Front. Meas. Biol. Divers*. **2010**, 194–207.
48. D. Schluter, M. W. Pennell, Speciation gradients and the distribution of biodiversity. *Nature* **546**, 48–55 (2017).
49. D. L. Rabosky, P. O. Title, H. Huang, Minimal effects of latitude on present-day speciation rates in New World birds. *Proc. R. Soc. B Biol. Sci.* **282** (2015).
50. J. Igea, A. J. Tanentzap, Angiosperm speciation cools down in the tropics. *Ecol. Lett.* **23**, 692–700 (2020).
